# Size-Dependent Permittivity for Alumina Powders

**DOI:** 10.3390/nano16070436

**Published:** 2026-04-01

**Authors:** Tien-Fu Yang, Hsien-Wen Chao, Bo-Wie Tseng, Yu-Syuan Dai, Tsun-Hsu Chang

**Affiliations:** 1 Department of Physics, National Tsing Hua University, Hsinchu 30013, Taiwan; yang168@gapp.nthu.edu.tw (T.-F.Y.); s9822817@m98.nthu.edu.tw (H.-W.C.); yusyuan@gapp.nthu.edu.tw (Y.-S.D.); 2 College of Semiconductor Research, National Tsing Hua University, Hsinchu 30013, Taiwan; tsengbowei@gapp.nthu.edu.tw

**Keywords:** dielectric measurement, permittivity manipulation, permittivity enhancement, hybrid model, surface-charge effect

## Abstract

Alumina is a commonly used ceramic material known for high permittivity, low dielectric loss, good thermal conductivity, and low cost. In the development of electronic devices, the size effect of powdery materials is crucial, particularly in applications involving composite materials. This study introduces the field-enhancement method (FEM) to measure the resonant frequency ($f_{0}$) and the quality factor (*Q*) of alumina powders packed in a Teflon container and placed on top of the central rod in the proposed cavity. The measured resonant condition ($f_{0}$ and *Q*) is mapped to a contour plot and simulated using a high-frequency structure simulator (HFSS). The contour mapping technique allows the researchers to obtain the effective complex permittivity of alumina–air composites. The complex permittivity of the alumina powder is retrieved using a hybrid model and the effective medium theories (EMTs), respectively. The Landau–Lifshitz–Looyenga (LLL) model is compared with the results using the hybrid model for its applicability. The dielectric constant and the loss tangent of the alumina powder are found to increase as the powder size reduces. A power relation is found to fit the obtained permittivity, covering sizes ranging from nanometers to micrometers, and a surface-charge scaling argument is proposed to explain the observed trend. This finding opens a new avenue for manipulation of permittivity in composite materials and has potential applications in stealth/absorber technology and as a self-limiter for grain growth during sintering.

## 1. Introduction

The dielectric properties of materials are essential for developing electronic and optoelectronic components in various applications [1,2,3,4,5,6,7,8]. The dielectric properties are also crucial in enhancing the electrical performance of semiconductor nano-fabrication [9]. The dielectric properties depend mainly on temperature $\left(T\right)$, frequency $\left(f\right)$, and electric field strength $\left(\left|E\right|\right)$. Debye relaxation refers to the relaxation response of a dielectric medium to an alternating electric field. The relaxation time of the medium closely relates to T [10,11]. The complex permittivity ($\varepsilon^{\prime }+{i\varepsilon }^{\prime \prime }$) of a dielectric material depends on the frequency of the applied electric field. Such a frequency-dependent effect is also known as dielectric dispersion, and the real and imaginary parts of the permittivity can be characterized using the Kramers–Kronig relations (KK relations) [12]. For some non-linear dielectrics, the permittivity relates to $\left|E\right|$. Known as the Kerr effect, this phenomenon is commonly observed in optical frequencies where the refractive index is proportional to the square of the electric field strength [13]. However, little research has been conducted on the size-dependent impact of powdery dielectrics.

Powdery dielectrics are commonly employed in producing composite materials for various emerging applications. The development of microwave devices relies heavily on high-k composites that exhibit a large real permittivity (dielectric constant) and low loss [14,15,16,17,18]. Dielectric films with high loss can be used for stealth technology and anti-reflection coatings [19]. Combining two or more materials with different volumetric fractions enables manipulation of the effective complex permittivity. Epoxy composites, which consist of a polymer host and dielectric/metal dopants, are commonly used in such applications [20]. Alumina powders are readily available in a range of particle sizes, from several hundred micrometers to a few tens of nanometers, and are cost-effective. Alumina is commonly utilized in electronic devices and can serve as a benchmark that links nano-sized particles to bulk materials. Furthermore, the alumina powders exhibit minimal particle–particle interactions, enabling a direct comparison with effective-medium theories (EMTs) [21,22,23,24,25].

It is essential to efficiently and accurately characterize the complex permittivity of powdery materials. The measurement of complex permittivity can be classified into two types: non-resonance [26,27,28,29,30] and resonance [31,32,33,34]. The non-resonant technique uses transmitted/reflected signals to extract the properties of the material under test (MUT). In contrast to the non-resonant approach, the resonant method, such as the perturbation method, only applies within a narrow bandwidth. However, it provides better resolution due to its strong electromagnetic coupling, making it useful for characterizing properties of low-loss dielectrics. In this study, the authors employ an improved resonant technique known as the field-enhancement method (FEM) [35]. The sample is positioned in the vicinity of the region with an enhanced electric field, which provides robust coupling between the sample and the resonant condition, namely the resonant frequency $f_{0}$ and the quality factor Q. The measured real permittivity can range from unity to several thousand by utilizing the contour mapping technique, and it can also be applied to materials with lossless to heavy loss properties [36]. Thus, the FEM with contour mapping is well-suited for the current study.

The characteristic of powdery dielectric is often influenced by its storage and processing conditions [37,38,39], as well as its particle size [40,41,42,43,44]. Typically, to characterize the permittivity of powdery materials, several composites with different volume fractions of the MUT are synthesized. From these measurements, the permittivity of MUT can then be extracted from established effective-medium theories (EMTs). However, due to different assumptions made during the development of these EMTs, it remains unclear which is the most applicable for the fabricated sample [45]. Additionally, until now, these theoretical EMTs have failed to obtain the imaginary part of the dopants. In 2021, Chao et al. developed a simulation-based effective medium model known as the hybrid model [46]. They discovered that it is well-suited for characterizing materials with irregular shapes, e.g., powder forms. This technique enables us to determine the complex permittivity of the MUT without fabricating composites with various volume ratios.

Alumina nanoparticles have become indispensable across a broad spectrum of industrial practices due to their unique combination of chemical stability, mechanical strength, and dielectric properties. In the energy sector, nano-alumina is a critical safety component in high-performance lithium-ion batteries [47]. It can also be used as a filler in polymer matrices to create thermal interface materials, because alumina possesses high thermal conductivity while remaining an electrical insulator. These composites facilitate efficient heat dissipation in miniaturized power electronics without compromising electrical safety [48]. Furthermore, in the field of electrical insulation, adding alumina nanoparticles to epoxy resins or mineral oils significantly improves volume resistivity and dielectric breakdown strength. These “nanodielectrics” are essential for high-voltage switchgear and transformer insulation, where they provide superior resistance to electrical aging compared with traditional bulk materials [49]. Additionally, alumina nanoparticles are key ingredients in modern flame-retardant formulations. When incorporated into polymers, they act as a physical heat-shielding barrier and a non-combustible char-former, significantly reducing the heat release rate and improving fire safety in aerospace and construction materials [50].

It is well-established that the complex permittivity of dielectric materials is frequency-dependent due to various polarization mechanisms, such as electronic, ionic, and interfacial (Maxwell–Wagner–Sillars) effects. While a broadband characterization would provide a complete dielectric spectrum, this study specifically focuses on the narrow-band behavior centered at approximately 2.45 GHz. This frequency was selected for two primary reasons: first, it is a critical ISM (Industrial, Scientific, and Medical) band widely used in microwave processing and sintering; second, the resonant field-enhancement method (FEM) utilized here offers the high sensitivity required to precisely detect subtle size-dependent shifts in low-loss alumina powders. By fixing the frequency, we isolate the influence of particle size and surface-to-volume ratios on the effective permittivity, providing a controlled baseline for understanding how these powders behave in high-frequency electromagnetic environments.

## 2. Materials and Methods

**Figure 1:**
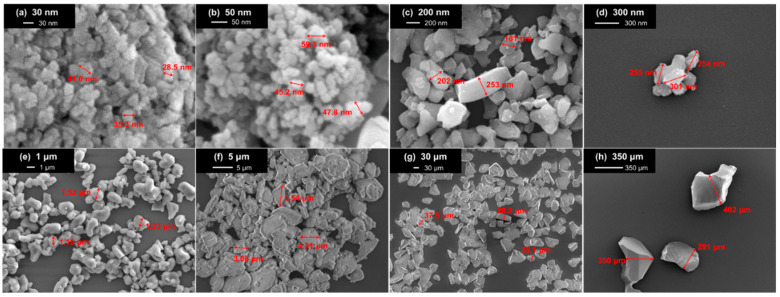
The SEM images of the alumina powders with the averaged size being (**a**) 30 nm, (**b**) 50 nm, (**c**) 200 nm, (**d**) 300 nm, (**e**) 1 μm, (**f**) 5 μm, (**g**) 30 μm, and (**h**) 350 μm.

The alumina powders in this study are purchased from two vendors, Taiwan Union Abrasives Corp., Hsinchu County, Taiwan (350 μm, 30 μm, 5 μm, 1 μm, 300 nm, and 200 nm) and US Research Nanomaterials Inc., Houston, TX, USA (50 nm and 30 nm). The purity of each powder exceeds 99%. The densities of the powders are 3.97 g/cm^3^. The averaged particle sizes are characterized using a scanning electron microscope (SEM) (JSM-7610F, JEOL, Tokyo, Japan). Figure 1 shows the SEM images of the samples. As shown in the photos, the samples are mostly ellipsoidal or irregular.

**Figure 2:**
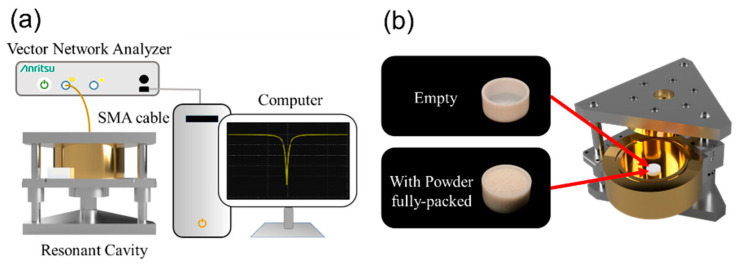
(**a**) Experimental setup. A test signal is sent from the Vector Network Analyzer (VNA) to a resonant cavity through an SMA coaxial cable. The computer is connected to the VNA to analyze the signal reflected by the cavity. (**b**) The sampled powder is packed into a 1 c.c. Teflon container to the fullest volume ratio and placed in the cavity’s center.

Figure 2a shows the experimental setup for material characterization. The Vector Network Analyzer (VNA) (Anritsu MS46122A, Kanagawa, Japan) injects a probing signal into the resonant cavity, and the reflected signal is examined through an SMA (SubMiniature version A) coaxial cable (Würth Elektronik, Niedernhall, Germany). The frequency response of the reflected signal is displayed on the computer for further diagnostics. From the measured reflection response, one can obtain the resonant frequency $f_{0}$ and the quality factor Q. Figure 2b illustrates how the samples are prepared and placed into the resonant cavity. The powders are packed into a 1 c.c. Teflon container to the fullest filling volumetric ratio to form air–powder composites. The 1.0 mL container has an inner radius of 7.0 mm and a height of 6.5 mm. The wall thickness of the container is 1.0 mm. The Teflon container with a fully packed sample was placed on top of the central metal rod within a resonant cavity [36].

The sample is positioned near an area with an enhanced electric field, resulting in a robust interaction between the sample and the resonant cavity. The FEM notably differs from the perturbation method in its approach. The resonant frequency $f_{0}$ and Q-factor measured through FEM are complex functions that are dependent on the sample’s complex permittivity. Using a full-wave simulator such as the high-frequency structure simulator (HFSS) (Ansys, Inc., Canonsburg, PA, USA), a contour map is generated to visualize the data.

**Figure 3:**
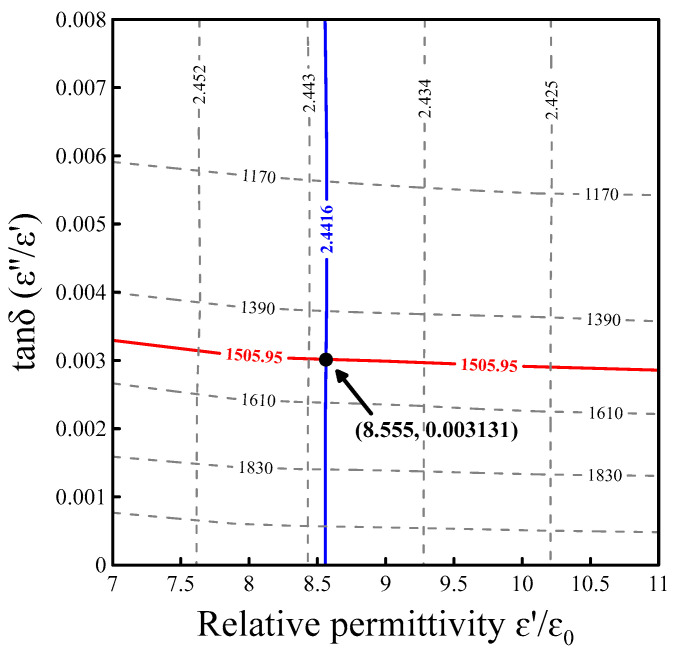
Contour map constructed by HFSS simulations. The X-axis and Y-axis show the relative permittivity and the dielectric loss tangent, respectively. With the frequency response (blue line) and quality factor (red lines) measured via the VNA, the relative permittivity and the dielectric loss tangent of the MUT can be found (the intersection), as shown in the figure.

Figure 3 shows the contour established by the HFSS simulation of the dielectric constants ranging from 7 to 11 and loss tangents ranging from 0 to 0.008. Two contour lines connect points of equal resonant frequency $f_{0}$ and Q-factor. The blue curve represents the measured resonant frequency $f_{0}$, and the red curve expresses the measured Q-factor. Their intersection uniquely determines $\varepsilon^{\prime }/\varepsilon_{0}$ and the loss tangent ($\tan\delta =\varepsilon^{\prime }/\varepsilon^{\prime \prime }$) of the sample [36]. The contour map can cover a broad range of complex permittivity in materials from a low to extremely high dielectric constant and from lossless to lossy dielectric properties.

The sample container is fully included in the HFSS simulation. Teflon was selected as the container material due to its low dielectric loss and near transparency at 2.45 GHz, minimizing its impact on the measurement. Detailed descriptions of the container modeling and material considerations can be found in Refs. [36,46].

It is worth noting that the authors employ two mesh methods in the full-wave simulation. One is the traditional method, i.e., considering the whole sample as a material with effective complex permittivity (${\varepsilon^{\prime }}_{eff}+i{\varepsilon^{\prime \prime }}_{eff}$). However, the sample is a composite material of alumina powder and air. To extract the complex permittivity of the alumina powder, we introduce another mesh method: a hybrid model [46]. The hybrid model was first introduced to simulate plastic material with irregular shapes. The hybrid mesh method (i.e., the hybrid model) reduces the amount of computer memory required, thereby shortening the simulation time while providing accurate results.

**Figure 4:**
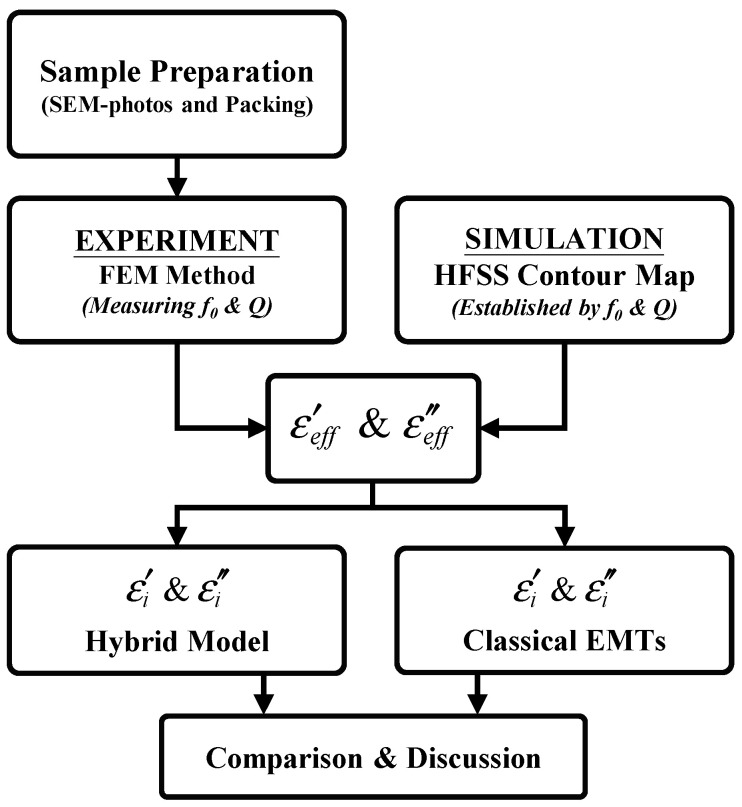
Flowchart of the current study.

Figure 4 displays the flowchart of the current study. First, the powder samples are examined using SEM (Figure 1); then, they are packed into a Teflon holder (Figure 2b) and measured using FEM (Figure 2a). By mapping the contour plot constructed using traditional HFSS simulation (Figure 3), one can obtain ${\varepsilon^{\prime }}_{eff}+i{\varepsilon^{\prime \prime }}_{eff}$. The authors employ the hybrid model and EMTs to calculate the complex permittivity of the inclusion (${\varepsilon^{\prime }}_{i}+i{\varepsilon^{\prime \prime }}_{i}$). Results from the four EMTs are first statistically compared with each other, and the best-fit result is then compared with those from the hybrid model.

## 3. Results and Discussion

**Figure 5:**
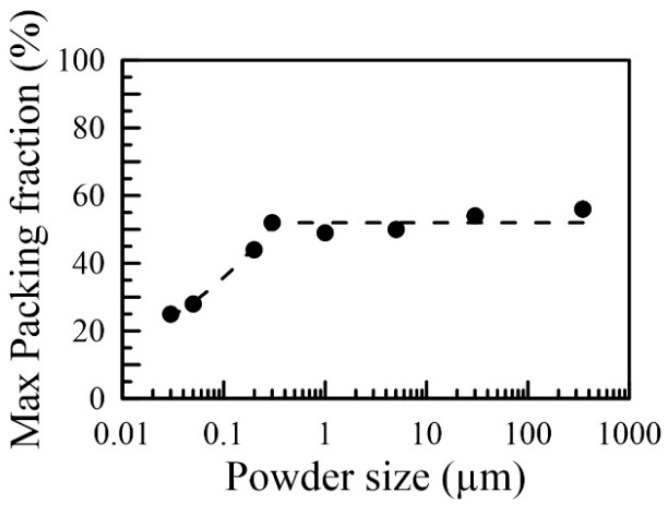
The maximum packing fraction of each powdery sample. The dashed line shows the behavior of the maximum packing fraction. Powders in micron sizes or larger exhibit roughly the same maximum packing fraction (~50%). Interestingly, the maximum packing fractions decrease when the particle sizes are smaller than 200 nm.

Figure 5 illustrates the maximum achievable packing fraction of alumina powder for different sizes. As the size of the powder increases to the micron range or above, the packing fraction tends to plateau at around 50%. The variations in the packing ratio could be attributed to differences in particle shape, as shown in Figure 1, or to the packing methods utilized [51]. When the particle size decreases to the nanoscale, for instance, 100 nm, the maximum packing fraction significantly decreases, suggesting that rearranging and packing the powders becomes more challenging [52]. This result is counterintuitive at first glance. One might expect the highest packing fraction to increase as particle size decreases, since finer powders can fill more vacancies.

As the size of the alumina powder decreases, the packing fraction decreases because smaller particles have a higher surface area-to-volume ratio than larger particles, which tend to be more irregular in shape; therefore, this further reduces their ability to pack efficiently. Additionally, as the particles become smaller, they are more likely to agglomerate or stick together, creating larger void spaces between clusters formed by neighboring particles. The decrease in packing fraction as the size of the alumina powder reduces is due to the larger spaces between particles, the irregular shape of the particles, and the tendency of the particles to agglomerate.

The MUT in this study can be treated as a composite of two materials. The inclusion is the particle under test, and the matrix (host) is the air. As shown in Figure 4, once ${\varepsilon^{\prime }}_{eff}+i{\varepsilon^{\prime \prime }}_{eff}$ is obtained, ${\varepsilon^{\prime }}_{i}+i{\varepsilon^{\prime \prime }}_{i}$ can be extracted. As shown in Figure 1, the shapes of the alumina powder are ellipsoidal or even irregular. The shape of the alumina powders can be simulated with the hybrid mesh method, which was shown to be effective for irregular plastics. Details of the hybrid method can be found in Ref. [46].

Four EMTs are applied. They are the Landau–Lifshitz–Looyenga model (LLL), Lichtnecker’s logarithmic mixing law (LOG), the Maxwell–Garnet model (MG), and the symmetric Bruggeman formula (sBM) [19]. These models are selected for their applicability to composites with low-contrast permittivity or irregularly shaped dopants, as reported in Ref. [22]. Detailed equations for the four theories can be found in the Supplementary Materials. Our study found that the LLL model best fits the measurement. It reads

(1) $${{\varepsilon^{\prime }}_{eff}}^{1/3}=v{{\varepsilon^{\prime }}_{i}}^{1/3}+\left(1-v\right){{\varepsilon^{\prime }}_{m}}^{1/3}$$

where v is the volume ratio of the dielectric (powder).

**Figure 6:**
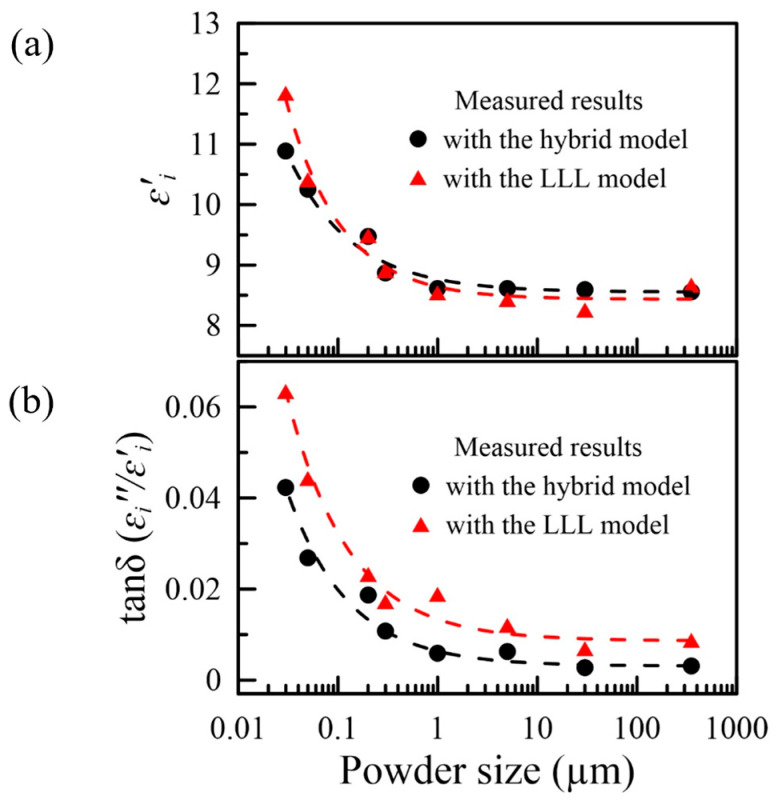
Comparison of the results from the hybrid and the LLL models. (**a**) The dielectric constant versus the powder size; (**b**) the dielectric loss tangent versus the powder size.

The measured effective complex permittivity is denoted as ${\varepsilon^{\prime }}_{eff,\;exp}$ for the real part and ${\varepsilon^{\prime \prime }}_{eff,\;exp}$ for the imaginary part. Using the formula of EMTs (LLL, LOG, MG, and sBM), the complex permittivity of the matrix (${\varepsilon^{\prime }}_{m}\;\&\;{\varepsilon^{\prime \prime }}_{m}$) and the inclusion (${\varepsilon^{\prime }}_{i}\;\&\;{\varepsilon^{\prime \prime }}_{i}$) are obtained. The fitting results are displayed in Figure S1. The left (right) y-axis shows the real (imaginary) part of the complex permittivity. MG and LOG models are observed to give relatively large predictions in the real part compared to the other models. By contrast, the imaginary part depends on the powder size. LOG and sBM models tend to provide larger predictions for nanoscale powder (30 nm and 50 nm), while for micron-sized powder, MG and LOG are more effective. The details of the results obtained are listed in Table S1. The predicted values from the LLL model are compared with those from the results of the hybrid model, as shown in Figure 6. The permittivity is fitted as $r^{-0.79}$ (LLL model) with $R^{2}=0.9768$ and RMSE=0.1614, and $r^{-0.68}$ (the hybrid model) is fitted with $R^{2}=0.9748$ and RMSE=0.2326, where r is the radius of the particle.

It is important to note that the permittivity values assigned to the alumina particles in Figure 6 are effective quantities extracted under the assumption that the particle is homogeneous. If a size-dependent surface layer exists, these values represent an average over the particle volume. Therefore, the observed size dependence may reflect a changing volume-to-surface ratio rather than a change in the bulk material properties.

Figure 6 compares the measured complex permittivity retrieved with the two models. They exhibit the same trends. For the real part of the complex permittivity of the inclusion (as shown in Figure 6a), the two models are consistent with the powder size in the micro-scale or larger, and they differ as the powder size gets smaller. Unlike the real part, the imaginary part, or the dielectric loss tangent, the values predicted with the two models differ significantly. As reported, the EMTs tend to produce poor predictions for the imaginary part [45]. Therefore, the hybrid model seems to provide a reliable result, though both have the same trend. Figure 6 also shows that when the particle size becomes smaller and enters the nanoscale, the relative permittivity and the dielectric loss tangent tend to increase.

Measuring uncertainty in the extracted complex permittivity originates from the precision of the primary observables (*f*_0_ and *Q*) and the physical dimensions of the sample. The Vector Network Analyzer (VNA) provides a frequency resolution of 1 Hz, and the resonant peak *f*_0_ is stable within ±0.5~1 MHz. The Q factor, determined by the 3 dB bandwidth, carries an estimated relative uncertainty of ±5%. The uncertainty in the powder packing fraction is estimated at ±2%, primarily due to the ±0.1 mm tolerance in measuring the sample filling height within the Teflon holder. Using the contour mapping method (as validated in Ref. [36]), we performed a sensitivity analysis by varying these inputs. The resulting experimental uncertainty for real permittivity is approximately ±3%, and for the loss tangent, it is approximately ±8%.

The observed increase in both the real and imaginary parts of the permittivity of alumina particles as their size decreases below ~200 nm is significant and calls for physical interpretation. While a complete microscopic theory is beyond the scope of this work, two complementary hypotheses can be considered. For a dielectric particle much smaller than the wavelength, the quasistatic polarizability is proportional to its volume ($P_{paricle}\propto r^{-3}$). However, when a large number of such particles are packed into a composite, the total interfacial area between the particles and the air matrix scales as $r^{-1}$ for a fixed volume fraction ($P_{compsite}\propto r^{-3}\times r^{2}=r^{-1}$). Each interface carries bound surface charges under an applied field, and these surface charges contribute to the macroscopic polarization. A simple scaling argument can be made: if each particle contributes a surface dipole moment density proportional to its surface area, the effective polarization of the composite also becomes $r^{-1}$. This leads to an effective permittivity that increases as particle size decreases, consistent with the power-law trend in Figure 6a ($r^{-0.79}$ for the LLL model and $r^{-0.68}$ for the hybrid model).

The $r^{-1}$ dependence also suggests that the dielectric response is dominated by surface-layer polarization mechanisms, effectively treating the surface state as an intrinsic variable of the nanoparticle system. This geometric effect does not imply that the intrinsic permittivity of the alumina material itself changes; rather, the measured permittivity of particles (as extracted by the hybrid model) incorporates the enhanced surface polarization because the model treats each particle as a homogeneous inclusion. A nanoscale particle may be better described as a core–shell system with a surface layer with different dielectric properties from the bulk. The hybrid model, however, lumps this surface contribution into an apparent bulk permittivity.

The increase in tanδ with decreasing size is more difficult to explain from purely geometric arguments. A plausible cause is the higher density of defects, dangling bonds, or adsorbed water on the large specific surface area of nanoparticles. These defects can act as relaxation centers at microwave frequencies, increasing the imaginary part of the permittivity.

Alternatively, increased loss may originate from Maxwell–Wagner–Sillars (MWS) interfacial polarization, which becomes more pronounced when the particle size is reduced, and the total interfacial area grows. The MWS effect produces a low-frequency relaxation that can extend into the microwave range if the conductivity contrast is sufficient [53]. The measurement system uses a network analyzer (Anritsu MS46122A) with an output signal of only 1 mW. This low power level is standard for diagnostic measurements and is generally insufficient to cause macroscopic thermal effects in ceramic or plastic samples. At the nanoscale, the high surface-to-volume ratio enhances the density of bound surface charges and defects, which act as centers for Maxwell–Wagner–Sillars (MWS) interfacial polarization. This interpretation is consistent with recent studies on oxide-filled nanocomposites, where the presence of nanoparticles has been shown to significantly modify dielectric behavior through interphase-related mechanisms [54].

At present, our data cannot distinguish between these mechanisms. Further experiments, such as controlled annealing to remove adsorbates or measurements at cryogenic temperatures, would be needed to clarify the origin of the loss.

**Figure 7:**
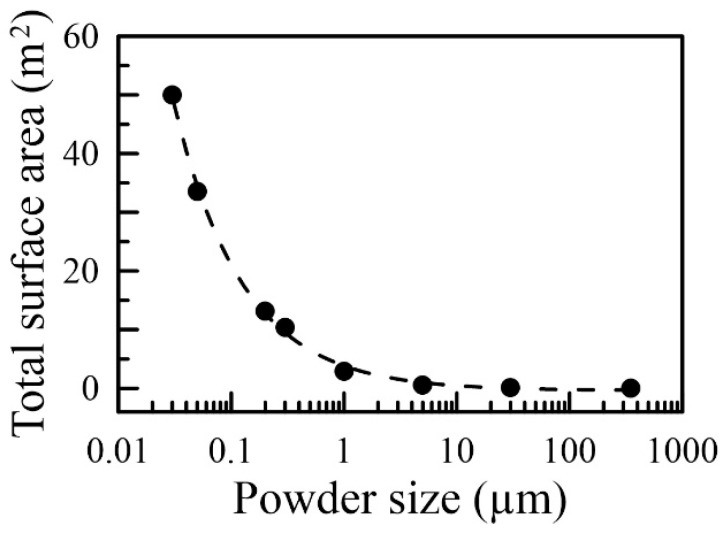
The total surface area of the sampled powder considering the fullest packing ratio. The dashed line is a power-law fit with exponent −0.70 (*R*^2^ = 0.9980, *RMSE* = 0.0884).

Figure 7 illustrates the total surface area of packed powder with the maximum packing fraction. When the powder size becomes smaller than 1 μm, the entire surface area of the packed powder tends to increase significantly. The total surface area scales as approximately $r^{-0.70}$ (with an R-squared of 0.9980 and RMSE of 0.0884), which is consistent with the result from the hybrid model. This agreement indicates the applicability of the simulation-based hybrid model for the powder and indicates the effect of increasing polarization due to the small-size effect, which is profound for the applications of composite materials.

## 4. Conclusions

Characterizing the complex permittivity of the powders is challenging. This work incorporated several techniques. First, FEM and the contour mapping technique were used to obtain ${\varepsilon^{\prime }}_{eff}+i{\varepsilon^{\prime \prime }}_{eff}$ for the alumina–air composites. Then, ${\varepsilon^{\prime }}_{i}+i{\varepsilon^{\prime \prime }}_{i}$ was obtained for alumina powder in various particle sizes with the hybrid model and the effective medium theories. Four effective medium theories were examined, where the LLL model agrees well with the measurement and is consistent with the hybrid model.

The observed size-dependent effects for the complex permittivity of alumina nanoparticles are strongly correlated with the total surface area of the powder compact. This suggests that surface-related polarization mechanisms dominate at the nanoscale. A simple scaling argument based on surface dipole density reproduces the power-law trend for the real part. The accompanying increase in the loss tangent may be attributed to defect-induced relaxation or interfacial polarization, but this requires further investigation to determine the exact mechanism. The results demonstrate that the effective permittivity of alumina powder can be manipulated over a wide range simply by controlling the particle size—a finding with immediate practical implications for composite materials.

Reducing the particle size raises both the real and imaginary parts of the complex permittivity of the powdery material. Such an intriguing phenomenon has potential in stealth/absorber technology and allows one to manipulate the complex permittivity of the composite materials. Furthermore, the high loss tangent at the nanoscale might be used to sinter the materials without a susceptor and serve as a self-limiter for the grain growth during sintering. This finding is associated with the alumina powder; however, it could be applied to other powdery materials with weak or no particle-particle interaction. In addition, the theoretical limit of the permittivity for even smaller particles is under further investigation.

While the current results clearly demonstrate a size-dependent trend at 2.45 GHz, characterized by an $r^{-1.00}$ scaling law, further investigation into higher-order harmonics of the resonant cavity could reveal how this size effect evolves across different frequency ranges. Future studies utilizing multi-mode resonance mapping will be instrumental in determining if the increase in observed permittivity is linked to specific relaxation frequencies that shift with particle size. Nonetheless, the findings presented here provide a vital empirical foundation for the design of alumina-based nanocomposites and absorbers operating in the S-band.

## Data Availability

The data that support the findings of this study are available from the corresponding author upon reasonable request.

## Supplementary Materials

The following supporting information can be downloaded at https://www.mdpi.com/article/10.3390/nano16070436/s1. Figure S1: The experimental results and predictions from the EMTs (LLL, LOG, MG, and sBM) were compared for alumina samples of different sizes: (a) 30 nm, (b) 50 nm, (c) 200 nm, (d) 300 nm, (e) 1 μm, (f) 5 μm, (g) 30 μm, and (h) 350 μm; Table S1: Complex permittivity retrieved from the EMTs and the calculated sums of all deviation squares.
