# Supplementary material for: Size-Dependent Permittivity for Alumina Powders
Source: Nanomaterials (Basel). 2026 Apr 1;16(7):436. doi: 10.3390/nano16070436 (PMC13074779; doi:10.3390/nano16070436)
Supplement: Supplementary file 1 [file nanomaterials-16-00436-s001.zip › nanomaterials-4218588-supplementary.pdf]

Size-dependent permittivity for alumina powders

Tien-Fu Yang, Hsien-Wen Chao, Bo-Wie Tseng, Yu-Syuan Dai, and Tsun-Hsu Chang

Supplementary Materials

In this study, we utilized the simulation-based hybrid model to investigate the complex permittivity of powdery alumina with varying particle sizes. We compared our findings with those obtained from four theoretical EMTs: the Landau-Lifshitz-Looyenga model (LLL) (S1), Lichtnecker's logarithmic mixing law (LOG) (S2), Maxwell-Garnet model (MG) (S3), and symmetric Bruggeman formula (sBM) (S4). These models were selected based on their ability to be applied to composites with low-contrast permittivity or irregularly-shaped dopants, as previously reported.[22]

$$\varepsilon_{eff}^{1/3} = f\varepsilon_i^{1/3} + (1-f)\varepsilon_m^{1/3} \tag{S1}$$

$$\varepsilon_{eff} = f\log\varepsilon_i + (1-f)\log\varepsilon_m \tag{S2}$$

$$\varepsilon_{eff} = \varepsilon_m + 3f\frac{\varepsilon_i - \varepsilon_m}{\varepsilon_i + 2\varepsilon_m - f(\varepsilon_i - \varepsilon_m)} \tag{S3}$$

$$\frac{\varepsilon_i - \varepsilon_{eff}}{\varepsilon_i + 2\varepsilon_{eff}}f + \frac{\varepsilon_m - \varepsilon_{eff}}{\varepsilon_m + 2\varepsilon_{eff}}(1-f) = 0 \tag{S4}$$

where f is the volume ratio of the inclusion (powder), and $\varepsilon_{eff}, \varepsilon_i, \varepsilon_m$ are the relative permittivity of the effective medium, the inclusion (powder), and the matrix (air), respectively.

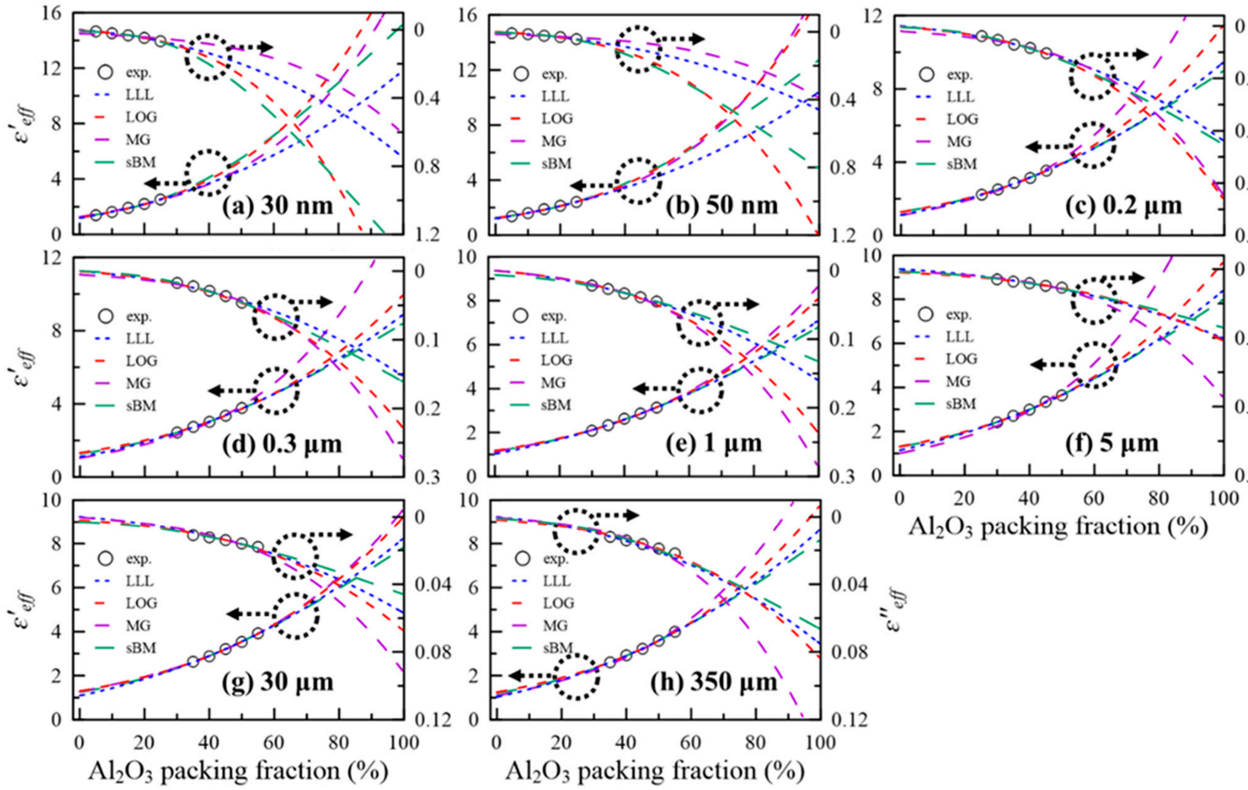

Figure S1. The experimental results and predictions from the EMTs (LLL, LOG, MG, and sBM) were compared for alumina samples of different sizes: **(a)** 30 nm, **(b)** 50 nm, **(c)** 200 nm, **(d)** 300 nm, **(e)** 1 µm, **(f)** 5 µm, **(g)** 30 µm, and **(h)** 350 µm. The real (imaginary) part of the effective complex permittivity under various packing fractions (x-axis) were shown on the left (right) y-axis.

Air-powder composites of different packing fractions were formed by compressing the powders into a container to determine the complex permittivity of both the matrix (ε_m' & ε_m'') and the inclusion (ε_i' & ε_i''). The effective complex permittivity of each composite is then determined using the contour mapping method, and the results are compared with predictions from the LOG, LLL, MG, and sBM EMTs. The left (right) y-axis of the graph in Figure S1 shows the real (imaginary) part of the complex permittivity. The MG and LOG models give relatively large predictions in the real part compared to the other two models. The imaginary part varies with the powder size, with the LOG and sBM models tending to provide larger predictions for nano-scale powders (30 nm and 50 nm), while the MG and LLL models become more accurate for micron-sized powders. The retrieved results are summarized in Table S1.

$$\begin{cases} \Delta' = \sum\left(\varepsilon'_{eff,\mathrm{EMT}} - \varepsilon'_{eff,\mathrm{exp.}}\right)^2 \\ \Delta'' = \sum\left(\varepsilon''_{eff,\mathrm{EMT}} - \varepsilon''_{eff,\mathrm{exp.}}\right)^2 \end{cases}, \quad \text{(S5)}$$

which are listed in the last two columns of Table 1. From (S5), the four models fit the experimental results well ($\Delta' < 0.1 \& \Delta'' < 0.01$). However, the real and imaginary parts of the air are about 1.0006 and 0. The LLL model gives the predictions closest to the reference values. Therefore, the predicted values from the LLL model are used to compare with those from the simulation-based hybrid model as Figure 6 shows.

TABLE SI. Complex permittivity retrieved from the EMTs and the calculated sums of all deviation squares.

		Powder Size (μm)							
	EMT	350	30	5	1	0.3	0.2	0.05	0.03
ε_i'	LLL	8.665	8.253	8.430	8.534	8.909	9.484	10.41	11.84
	LOG	9.788	9.256	9.727	9.706	9.996	11.53	17.22	23.35
	MG	12.02	9.610	15.06	14.03	14.86	16.20	18.04	100.0
	sBM	8.177	7.836	8.015	8.247	8.419	9.009	12.75	14.19
ε_i''	LLL	0.0755	0.0571	0.1013	0.1606	0.1535	0.2202	0.4612	0.7503
	LOG	0.0842	0.0678	0.1047	0.2373	0.2300	0.3327	1.2101	1.7599
	MG	0.1407	0.0923	0.1883	0.2874	0.2767	0.3259	0.3912	0.6111
	sBM	0.0667	0.0461	0.0854	0.1326	0.1619	0.2271	0.8100	1.3123
ε_m'	LLL	1.032	1.089	1.017	1.029	1.065	1.106	1.033	1.041
	LOG	1.248	1.296	1.318	1.169	1.326	1.276	1.218	1.209
	MG	1.088	1.279	1.010	1.130	1.047	1.103	1.231	1.265
	sBM	1.206	1.253	1.306	1.154	1.278	1.306	1.268	1.264
ε_m''	LLL	$< 10^{-12}$	$< 10^{-4}$	$< 10^{-4}$	$< 10^{-9}$	$< 10^{-11}$	$< 10^{-4}$	0.0004	$< 10^{-4}$
	LOG	0.0018	0.0020	0.0051	0.0004	0.0004	0.0019	$< 10^{-13}$	0.0031
	MG	0.0004	0.0003	0.0033	0.0003	0.0052	0.0099	0.0147	0.0217
	sBM	0.0011	0.0030	0.0039	0.0065	$< 10^{-10}$	0.0022	0.0037	0.0074
Δ'	LLL	0.0009	0.0017	0.0017	0.0024	0.0023	0.0022	0.0020	0.0003
	LOG	0.0006	0.0007	0.0030	0.0041	0.0030	0.0030	0.0039	0.0013
	MG	0.0221	0.0007	0.0925	0.0041	0.0523	0.0027	0.0046	0.0109
	sBM	0.0010	0.0017	0.0019	0.0025	0.0022	0.0025	0.0073	0.0042
Δ''	LLL	$< 10^{-4}$	$< 10^{-5}$	$< 10^{-5}$	$< 10^{-5}$	$< 10^{-4}$	$< 10^{-5}$	$< 10^{-5}$	$< 10^{-5}$
	LOG	$< 10^{-5}$	$< 10^{-5}$	$< 10^{-5}$	$< 10^{-4}$	$< 10^{-4}$	$< 10^{-5}$	$< 10^{-4}$	$< 10^{-5}$
	MG	$< 10^{-4}$	$< 10^{-4}$	$< 10^{-4}$	$< 10^{-4}$	$< 10^{-4}$	$< 10^{-4}$	0.0002	0.0007
	sBM	$< 10^{-5}$	$< 10^{-5}$	$< 10^{-5}$	$< 10^{-5}$	$< 10^{-5}$	$< 10^{-5}$	$< 10^{-4}$	$< 10^{-4}$
